# Salty and Savoury Snacks Compliance with 2016 and 2019 Sodium Content Targets—Durban Market, South Africa

**DOI:** 10.3390/ijerph192114118

**Published:** 2022-10-29

**Authors:** Nomcebo Zama, Kemlall Ramdass, Kgabo Mokgohloa

**Affiliations:** 1 Department of Management Sciences, Operations and Quality Management, Durban University of Technology, 49 ML Sultan, Greyville, Durban 4001, South Africa; 2 Department of Industrial Engineering, University of South Africa, 23 Pioneer Road, Florida 1958, South Africa

**Keywords:** sodium content, salty, savoury snacks, South Africa

## Abstract

South Africans consume a significantly high amount of sodium from salty snacks. The study aimed to evaluate savoury snacks (ready-to-eat savoury snacks, flavoured potato crisps and flavoured ready-to-eat, savoury snacks and potato crisps—salt and vinegar only) for compliance with the June 2016 and 2019 target date for sodium reduction as set out by the Department of Health in Regulation 214. It also looked at low-sodium claims made by the evaluated products. The study’s research problem is located at the confluence of three critical trends: increasing consumption of sodium-containing salty snacks, increasing sodium-related disease burden and deaths and attempts to regulate sodium intake through regulation as a response. A total sample of 90 products belonging to the above categories was considered. Sodium content information was collected from the selected product packages. The study also applied the Association of Official Analytical Chemists’ (AOAC) official method 984.27 in laboratory tests to verify low-sodium claims on the sampled products. The study showed that out of the 90 selected snacks, 26% of the snacks did not meet their 2019 targets, while 4% did not meet their 2016 targets. Fisher’s exact tests showed that no snack category had a better inclination toward meeting 2019 tests than others. The laboratory tests showed that 4.4% of the products made a compliant low-sodium content claim (sodium levels below 120 mg Na/100 g), while one made a non-compliant sodium content claim. Among other things, the study recommended increased product compliance monitoring and evaluation, using standardised, rigorous sodium testing and measuring systems, using more consumer-friendly labels and consumer education on sodium labelling.

## 1. Introduction

Salt is a common everyday ingredient used to flavour and preserve food in households and commercial entities. Over the years, there has been increasing concern about the quantity of salt consumed by societies, including processed salty snacks [1]. Excessive amounts of salt in both its discretionary and non-discretionary forms are linked to many health problems, especially cardiovascular problems [2]. Hypertension is a global problem that increases the risk of heart attack, stroke, kidney failure, and blindness. It has been reported that 1.13 billion people have been diagnosed with hypertension [3]. High salt intake was identified as one of the contributing factors to hypertension.

Non-discretionary salt comes in processed foods, and consumers do not have immediate control over it except to reduce taking such foodstuffs [1]. Such processed foods constitute a significant element of modern diets and lifestyles, and replacing them is not always a preferable choice for consumers. Supranational, international, and local entities continuously endeavour to reduce foods’ salt or sodium content. In this vein, South Africa implemented the Regulations Relating to the Reduction of Sodium in Certain Foodstuffs and Related Matters (Regulation 214) of the Foodstuffs, Cosmetics and Disinfectant Act, 1972, published in the Government Gazette by the Department of Health [4]. These regulations targeted the reduction in non-discretionary salt in 13 food categories. This study looks at three product classes that fall into three categories regulated for sodium reduction by 2016 and 2019 to ascertain the degree of success in meeting these two targets as illustrated in Table 1. The study looks at “Savoury snacks, excluding salt-and-vinegar”, “Flavoured Potato Crisps, excluding salt-and-vinegar” and “Flavoured ready-to-eat savoury snack and potato crisps—salt and vinegar only”. Collectively, in this study, these are referred to as salty snacks or salty, savoury snacks.

### 1.1. Background

Salty snacks are a popular treat among people of all ages. Several global research reports published by independent research agencies highlight continuous growth trends in salty snack volumes and revenues. For instance, Grand View Research, a US research firm, reports that in 2021, the global salty snacks industry realised a total revenue of US$250 billion and on top of that, it is expected to continue on a growth trajectory until 2030. The reports point to this growth rate as “urbanisation and hectic lifestyles” that have seen some individuals and households even substituting traditionally cooked meals for snacks [5]. A different agency also projected significant revenue growth for salty snacks [6]. Another report by Markets and Markets (2022) projected the global salty snacks industry revenue to rise to US $263 billion by 2027, and this report attributes this positive growth to changing lifestyles. The impact of COVID-19, especially the “stay-at-home” options and restrictions significantly increased the volume of snacking in developed countries. At the same time, constrained supply chains forced price increases and constrained demand, yet the industry recorded significant positive performance [7].

The salty snack market is broad and diverse in South Africa. South Africa’s salty snack revenue for 2020 amounted to US $1345 billion and was projected to grow by 3.94% between 2021 and 2026. The same report cites another study by Knorr, a well-known vegetable flavour brand, which states that 70% of SA consumers snacked on salty snacks with potato chips being the most popular snacks [7]. The consumption of snacks increased by 43% between 2020 and 2021. As of the second quarter of 2021, salty snacks held a 23% market share of all snacks purchased and consumed in South Africa. This declined to 16.1% in the second quarter of 2021 [8]. In addition to sales volumes, revenue and market share growth related to salty snacks, five major retailers dominate this market: Shoprite, Pick n; Pay, Spar, Checkers and Woolworths. As of the second quarter of 2021, 31% of all salty snacks were distributed via Shoprite, followed by 19% through Pick n’ Pay, 16.3% through Checkers, 10.8% via Spar, and 1.4% via Woolworths [8]. Generally, statistics point to an ever-increasing consumption of salty snacks—a trend that is not expected to slow down any time soon.

In this environment characterised by increasing snacking, some studies and reports talk of significant increases in high sodium-related diseases and conditions. Between 2009 and 2019, the death burden and disability risks associated with increased sodium intake increased in both the developed and developing world [9]. The World Health Organization (WHO) reports that globally 4.1 million people die because of sodium-related deaths. The same report castigates poor dietary choices that include snacking on high-sodium foodstuffs as severe sodium-related death risk factors. In South Africa, sodium-connected deaths are rising round about the same time snacking on sodium-rich foodstuffs is increasing. A National Department of Health report puts South African hypertension prevalence at 33% of the adult population [10]. The WHO also reports a considerable prevalence of people worldwide dying from cardiovascular diseases. In 2016, 17.9 million people died from cardiovascular diseases [11].

In response to such problematic situations, the South African government implemented regulatory measures to limit sodium content in selected foodstuffs. The South African Department of Health released the regulation in March 2013 for implementation in June 2016 and June 2019 [4]. The regulations have been amended two times, and government notice 1071/2017 included updates on specific definitions, the method of testing for sodium analysis and updated food categories [12]. The last amendment regulation, Government notice 812/2019, was published in 2019 and included updated food categories for processed and raw meat sausages [13].

The main objective of the regulations was to decrease the salt level in certain foodstuffs. The National Department of Health established this regulation to monitor consumers’ use of non-discretionary salt in South Africa [4]. Salt Watch noted that effective coordinated marketing strategies to communicate to South Africans regarding the use of discretionary salt could aid in empowering consumers [14]. Therefore, food manufacturers must comply with the levels set out by the government. Some scholars recommend that consistent monitoring of food products in South Africa is vital to ensure industry compliance and evaluate the impact of reduced sodium in the diet [15]. Before these regulations, South Africa lacked a harmonised policy for regulating sodium. Reducing sodium content in South Africa to the mandated level will contribute to the decrease in hypertension prevalence. However, it will take some time for the full impact of regulatory sodium reduction to be felt [16].

### 1.2. Salty Savoury Snacks and Sodium Content

In many studies, snack foods are not well-defined, although researchers seem to describe them well [17]. Snacks can be classified either as sweet or savoury, while savoury ones can be further classified into salty snacks and spicy snacks [18]. The common components of savoury snacks were the use of a starchy or vegetable base, fat/oils and salt and spices rather than sugar as main flavourants [17,18]. Salty, savoury snacks use salt as the main flavourant and are classified under different names. Potato crisps are made from potatoes, while ready-to-eat extruded salty snacks are usually made from starches such as flour and corn as the main ingredients. While potato chips can be made through frying and baking, extruded snacks are made through the extrusion process [19]. These snacks can come out with a puffy or crunchy texture depending on the ingredient, temperature, and pressure combination [20].

Salt is made up of sodium and chloride and plays various important roles in the savoury snack manufacturing processes. These are [21,22]:

- Texture enhancement—Salt can be manipulated to give the desired snack texture when combined with water, fats, starches, and other substances. It helps with a firm texture that is required in most savoury snacks.
- Flavourant—Salt enhances the taste of snacks. Many snacks apply it as their primary flavour rather than as an additive (for example, salt and vinegar-flavoured chips).
- Flavourant enhancer—Salts also enhance how other flavours in a savoury snack can come out. They suppress bitterness while reducing sweetness, making flavours such as vinegar more testable.
- Nutrient source—As the body requires sodium as a nutrient, salt in savoury snacks also serves this purpose.
- Emulsifying agent—Salt helps to bind protein with other substances to avoid product crumbling or disintegration.
- Preservative—Salt reduces water content in snacks, limiting microbial activity and making products last longer on shelves.

The reasons for the popularity of salty snacks are their ease of accessibility, affordability, and storability [23]. These appeal to both the manufacturer and the consumer. An adult eats an average of 40 grams of savoury snacks in the United States daily. Some attribute the increasing consumption of savoury snacks to dietary shifts as societies move away from traditional, more stable diets toward highly processed, sugary, and savoury diets [24].

A common aspect of salty, savoury snacks is their affordability to the rich and the poor, making them a socially cross-cutting foodstuff [25]. One of the demographic groups considered major salty snack consumers were school children [24], and potato chips are the commonest of such snacks [26]. The effect of affordability and convenience of snacks for the school environment combined with limited knowledge of health risks, and pressure from colleagues and family experiences make school children highly vulnerable to the adverse effects of high sodium in savoury snacks [27].

## 2. Materials and Methods

### 2.1. Research Design

The study applied a quantitative observational descriptive research design in investigating the phenomenon of interest, specifically the levels of compliance with 2016 and 2019 sodium reduction targets in snacks and crisps. The objects of observation were:

- Ready-to-eat savoury snacks;
- Flavoured potato crisps;
- Flavoured ready-to-eat, savoury snacks and potato crisps—salt and vinegar only.

The observation method involved looking at and recording selected snacks’ sodium content as reported on nutritional guide labels on their packaging.

### 2.2. Sampling

The study sampled snacks and crisps that belonged in the three listed categories available for sale in Durban. Convenience sampling was used and involved collecting data from readily available products. Additionally, this convenience was applied in selecting retailers from which the products or samples were collected. The products for observation were purchased from five South African retail shops in Durban: Pick n Pay, Woolworths, Spar, Checkers and Shoprite stores. The *Global Powers of Retailing Report for 2020*, which Deloitte publishes, ranks 250 of the world’s most prominent retail groups; four were Shoprite, Spar group, Pick n’ Pay and Woolworths from South Africa. This highlights that the entities from which the selected products were acquired have a broad outreach to South African and Durban societies.

Only snacks from the three categories with a nutritional information panel were included. In total, 90 products were bought and divided into three subgroups as per the sodium regulation. The sample was 40 units for C1, another 40 for C2 and 10 for C3.

### 2.3. Data Collection

The data collection process involved three steps. The first was the identification and collection of the actual products from the five retailers. A data collection checklist was used to ensure that products meeting the sample selection criteria were purchased and that there was no exclusion and duplication of eligible products and the inclusion of non-qualifying products. Once the samples were collected, sodium composition data recorded in mg Na/100 g were collected and recorded on a Microsoft Excel sheet.

### 2.4. Sodium Analysis in the Laboratory

The samples were analysed using the Association of Official Analytical Chemists (AOAC) official method 984.27. This test was used because of its availability to the researcher and its common use and acceptability as a reliable scientific testing method for elements in foodstuffs and beverages. The test uses the inductively coupled plasma-atomic emission spectroscopy (ICP-AES) principle [28].

The information collected from the nutritional table and artwork panel was captured on a spreadsheet by the researcher, and the information was separated into three specific categories. The data were entered two times to minimize errors. The researcher coded the results to ensure that no brand names were mentioned. Data were analysed using Microsoft Excel and SPSS version 23.

### 2.5. Quantitative Data Analysis

Descriptive statistics, mainly the mean or average scores and their accompanying standard deviations, were used for the tests. Kurtosis and skewness between −2.5 and 2.5 [29,30] were used as benchmarks to indicate that data were distributed in a manner that enabled it to be reliably analysed using mean scores.

Given the small sample size, statistical measures involving significance-level tests were considered a potential risk to research outcomes [31]. Fisher’s exact test was therefore used for further data analysis because of its suitability for small sample tests of associations [29].

### 2.6. Reliability and Validity

The research process noted the importance of reliability and validity. In this study, validity related to the extent to which the study measured what it intended to measure. To reiterate, this was the levels of sodium per 100 g in three types of salty snacks and to measure the actual sodium content in salty snacks with low sodium claims. To ensure the reliability of measurements, the research relied on the following:

- Cross-checking to ensure that the measured product belongs to the class they were assigned using a checklist;
- Measuring and recording each sample twice as a way of controlling data inputting errors;
- Only sampling salty snacks with explicit sodium content presented in milligrams per 100 g.

For the second observation, which involved laboratory measurement of nutrition content claims, the following measures were taken to enhance the study’s ability to measure what it intended to measure:

- The use of proper (ICP-AES) procedure as per the Association of Official Analytical Chemists (AOAC) official method 984.27 procedure;
- The repetition of tests to recheck outputs and investigate differences.

The study also reviewed the measurement methods that were applied in previous studies, including Hattingh [32], Peters et al. [15] and Korff [33], among others. This review identified strengths and weaknesses, which were applied to better the study’s methods. The above measures, as expected, improved the study’s validity.

## 3. Results

In a total sample, 96% of the varieties met their 2016 Na/100 mg targets, while only 4% did not meet their targets. In the same sample, 74% met their 2019 target, while 26% did not. Table 2 summarises all categories’ performance against benchmarks or targets for 2016 and 2019.

In the C1 category, 7% of the 40 varieties sampled did not meet their 2016 target of 800 mg/100 g, while 27% did not meet their 700 mg/100 g target for 2019. In the C2 category, all 40 varieties met their 2016 target of 650 mg/100, while 20% did not meet their 2019 target of Na 550 mg/100 g. In the C3 category, nine varieties or 90% met their 2016 target of Na 1000 mg/100 g, while 40% of the 10 varieties did not meet their 2019 target of Na 850 mg/100 g. C2 had the highest proportion (80%) of varieties meeting their 2019 targets, which was followed by C1 (73%) and C3 (60%). Comparatively, C2 had the highest proportion of varieties that met their 2016 target. This was followed by C1 (93%) and C3 (90%). C3 varieties lagged behind the other classes in terms of meeting the set sodium content targets.

Ready-to-eat savoury snacks (C1) had a mean of 532 (sd = +/−279, a minimum sodium content of 5 mg Na/100 g and a maximum of 1113 mg Na/100 g. Flavoured potato crisps (C2) had a mean of 507 (sd = +/−69), a minimum sodium content of 325 mg Na/100 g and a maximum of 630 mg Na/100 g. Finally, the C3 category (flavoured ready-to-eat, savoury snacks and potato crisps—salt and vinegar only) had a mean of 799 (sd = +/−189), a minimum sodium content of 512 mg Na/100 g and a maximum was 1036 mg Na/100 g. The kurtosis and skewness alphas for all three ranged from 1.0 to −1.0, indicating closeness to normal distribution in the data and therefore a high reliability of using mean scores to describe the data [34].

In terms of mean sodium content, this study’s 2019 sodium content mean of 798.6 differs from Korff’s (2019) mean of 884.33. This indicates a comparatively lower albeit still concerning level. The next section presents the individual results of the three categories starting with C1.

### 3.1. Ready to Eat Savoury Snacks (C1)

Table 3 summarises the observed descriptive statistical values for all snacks including ready-to-eat snacks presented under the C1, C2 and C3 categories.

For the C1 category, the mean Na mg/100 g for 2016 was 494 (sd = +/−249). The minimum sodium content was 5 mg/100 g recorded on Savoury Snack 2 and Savoury Snack 3, and the maximum was 793 mg/100 g recorded for Savoury Snack 20. Three (3) of the 40 varieties failed to meet their 2016 target, and their mean was 1010 (sd = +/−179). These were Savoury Snack 24, Savoury Snack 25 and Savoury Snack 29.

The mean Na mg/100 g for 2019 was 424 (sd = +/−237). The minimum sodium content was 5 mg/100 g recorded on Savoury Snack 2 and Savoury Snack 3, and the maximum was 595 mg/100 g recorded for Savoury Snack 39. Eleven (11) of the 40 varieties failed to meet their 2019 target. Their mean was 817 (sd = +/−150). Their highest sodium content per 100 g was 1113 mg/100 g and was for Savoury Snack 24 and Savoury Snack 25.

The kurtosis and the skewness for mg Na/100 g for 2016 and 2019 were below 2.5. For 2016, the kurtosis was 0.49 and the skewness was −0.9, while for 2019, the kurtosis was −0.8 and the skewness was at −0.99. This shows nearness to a normal distribution [35].

Amongst C1 snack varieties, five reported a nutrient content claim for sodium which was “low in sodium”. Their mean score was 103 (sd = +/−105) and their minimum and maximum sodium content was 5 mg Na/100 g and 270 mg Na/100 g, respectively.

### 3.2. Flavoured Potato Crisps (C2)

Table 3 above also summarises the observed descriptive statistical values for flavoured potato crisps also presented as the C2 category.

For varieties that met the 2016 target, the mean Na mg/100 g was 507 (sd+/−69). They had a minimum sodium content of 325 mg/100 g recorded for Flavoured Potato Crisp 5 and a maximum of 630 mg/100 g recorded for Flavoured Potato Crisp 25. None of the C2 varieties failed to meet their 2016 target of Na 650 mg/100 g.

For flavoured potato crisp varieties that met the 2019 target of 550 mg/100 g, the mean Na (mg)/100 g was 485 (sd = +/−58). The minimum sodium content was 325 mg/100 g recorded on Flavoured Potato Crisp 5, and the maximum was 550 mg/100 g recorded for Flavoured Potato Crisp 11, Flavoured Potato Crisp 12, Flavoured Potato Crisp 14 and Flavoured Potato Crisp 26. Thus, 12.5% or four out of 32 varieties were positioned at the 550 mg/100 g benchmark.

The eight C2 varieties that failed to meet the 2019 target of Na 550 mg/100 g had a mean of 593 (sd = +/−28). This translates to 8% above the 550 mg/100 g target for 2019. The variety with the lowest above-benchmark Na content was Flavoured Potato Crisp 20 with 557 mg/100 g. In the C2 group that failed to meet the 2019 target, the highest sodium content was 630 mg/100 g recorded for Flavoured Potato Crisp 25.

The kurtosis and the skewness for mg Na/100 g for 2016 and 2019 were below 2.5. For 2016, the kurtosis was 0.2 and the skewness was −0.5; for 2019, the kurtosis was 0.8 and the skewness was at −1. This shows nearness to a normal distribution [34].

### 3.3. Flavoured Ready-to-Eat, Savoury Snacks and Potato Crisps—Salt and Vinegar Only (C3)

Table 3 shows the observed descriptive statistical values for flavoured ready-to-eat, savoury snacks and potato crisps with salt and vinegar also presented as the C3 category.

In the C3 category, varieties that met the 2016 target had a mean of Na mg/100 g of 722 (sd = +/−179). Their minimum sodium content was 512 mg/100 g recorded on Salt and Vinegar Snack 3, and the maximum was 987 mg/100 g recorded for Salt and Vinegar Snack 6 and Salt and Vinegar Snack 7.

Only one C3 variety failed to meet the 2016 target, and this was Salt and Vinegar Snack 1 with a sodium content of 1036 mg/100 g. In the same category, varieties that met the 2019 target of 850 mg/100 g had a mean Na (mg)/100 g of 686 (sd = +/−58). Their minimum sodium content was 512 mg/100 g recorded for Salt and Vinegar Snack 3, and the maximum was 850 mg/100 g recorded for Salt and Vinegar Snack 5.

The mean for the four C3 varieties that failed to meet the 2019 target of 850 mg/100 g was 968 (sd = +/−75). This translates to 14% above the 850 mg/100 g target for 2019. The variety with the lowest above-target content was Salt and Vinegar Snack 4 with 861 mg/100 g. The highest sodium content was 1036 mg/100 g recorded for Salt and Vinegar Snack 1, and this translates to 22% sodium content above target.

The kurtosis and the skewness for mg Na/100 g for 2019 and 2016 were below 2.5. For 2016, the kurtosis was −1.44 and the skewness was −0.33; for 2019, the kurtosis was −2.47 and the skewness was at 0.07. This shows nearness to a normal distribution, suggesting the mean score’s reliability [34].

### 3.4. Varieties Failing to Meet Both 2016 and 2019 Targets

Table 4 summarises varieties that failed to meet both 2016 and 2019 targets which constituted four out of the 90 varieties studied (4.4%).

Of the four, three were from the C1 category (Savoury Snack 24, Savoury Snack 25, and Savoury Snack 29) and one was from the C3 category (Salt and Vinegar Snack 1). A study conducted in South Africa which collected sodium content using nutritional labels identified that categories which did not meet the legislation sodium levels as per R214 were potato crisps (41%) and salt and vinegar-flavoured snacks (42%) [15]. The study by Peters et al., therefore, highlighted that meeting the regulated sodium content target was, to an extent, a challenge to some manufacturers in the snacks and crisps business—findings that closely resonated with this study [15].

### 3.5. Low-Sodium Claims versus Laboratory Analysis Results

C1 was the only category with products with a “low in sodium” claim [36]. Products with such a claim were further studied in the laboratory to compare the sodium content labelled on the packaging versus the tested or scientifically detected levels. Savoury Snack 1, Savoury Snack 2, Savoury Snack 3, Savoury Snack 4 and Savoury Snack 14 were subjected to these tests.

Of the five, only Savoury Snack 4 had a lower than labelled sodium content of 9.47 mg Na/100 g versus the labelled 24 mg Na/100 g. Other snacks had lower sodium content on their labels than those detected in the laboratory. For instance, Savoury Snack 14 had a low sodium claim of 270 mg Na/100 g versus a detected content of 380 mg Na/100 g. Savoury Snack 1 had a claim of 112 mg Na/100 g and a detected content of 118 mg Na/100 g, while Savoury Snack 2 had a claim of 112 mg Na/100 g and a detected 118 mg Na/100 g sodium content.

## 4. Discussion

### 4.1. Ready-to-Eat Savoury Snacks (C1)

Generally, this study found comparatively lower minimum, maximum and mean sodium contents than Hattingh, who found a mean sodium content of 764 mg Na/100 g and 720 mg Na/100 g for March 2014 and March 2015 in RTE snacks, excluding salt and vinegar [32]. These were relatively low compared to the mean sodium content of only RTE snacks that did not meet the 2016 sodium content target. This study found a mean of 1009.67 mg Na/100 g among snacks that did not meet the 2016 target and 816.91 mg Na/100 g among those that did not meet the 2019 targets. In terms of the mean for all RTE snacks, regardless of whether they met the set targets or not, Hattingh’s [32] study found higher mean scores (764 and 720) compared to this study’s 532.33 (+/−278.79). Therefore, the Durban findings represent an improvement from Hattingh’s findings, although it must be noted that the two studies were conducted in different contexts.

Other studies also show similar trends where the average sodium content per snack category was higher than WHO guidelines. The NIHI study for Australia and New Zealand showed that only potato crisps had sodium content below 600 Na mg/100 g considered safe by the WHO. This was attributable to the fact that in New Zealand and Australia, the 2019 sodium reduction targets for extruded snacks and salt and vinegar chips were set above the WHO recommended levels. Thus, salt and vinegar chips had a target of 810 Na mg/100 g in Australia and 740 Na mg/100 g for New Zealand [37].

### 4.2. Flavoured Potato Crisps (C2)

For C2, in another study, a minimum sodium content of 175 mg Na/100 g and a maximum of 1670 mg Na/100 g were found in potato crisps [15]. They also found a mean of 721 on the same product out of a sample of 96. As highlighted earlier, C2 had the highest proportion (80%) of varieties meeting their 2016 targets. It was possible to replace salt with 30% potassium chloride and monosodium glutamate in a potato chips recipe without affecting the taste profile of the potato chips [38]. Another study in South Africa analysed food products included in the sodium regulation and reported that 70% of the flavoured potato crisps, excluding the salt-and-vinegar group, did not comply with the targets set out in 2016 and 2019, respectively [39]. This study, however, shows significantly lower non-compliance levels, which the researcher attributes to the data being collected closer to the deadlines when manufacturers had had enough time to reformulate their products. In addition, in the research of Swanepoel et al., the 2019 target was considered a futuristic target, and manufacturers were not compelled to meet it then [39]. Therefore, there was no pressure to rework product constituents to meet this future deadline.

Hattingh’s study also showed a significantly high mean sodium content for flavoured potato crisps as of March 2015 compared to the results obtained in this study [32]. The mean mg Na/100 g for potato crisps was 819 mg Na/100 g in 2015 compared to 506.68 for this study. While the comparison is complicated by timing and sampling differences, the baseline is that the current results indicate a generally lower level of sodium in potato crisps.

### 4.3. Flavoured Ready-to-Eat, Savoury Snacks and Potato Crisps—Salt and Vinegar Only

In the C3 category, varieties that met the 2016 target had a mean of mg Na/100 g of 722 (sd = +/−179). Hattingh’s study found a considerably higher mean sodium content in salt and vinegar ready-to-eat, savoury snacks and potato crisps than in this study [32]. These were 1330 and 1149 mg Na/100 g for data collected in March 2014 and 2015, respectively. This was much higher than the mean sodium content in this study’s products, which did not comply with the 2016 and 2019 targets. The comparison suggests higher sodium contents in the past than in the present, although it is vital to comment that Hattingh’s study sampled two popular retailers in South Africa, while this study sampled stores in Durban

### 4.4. Compliance by Product Category

The data indicate that most snacks had sodium levels below the 2019 target on average when studied as a group. However, when the minimum and maximum Na/100 mg were considered, some snacks contained excessive amounts of sodium—far above the 2019 regulated levels. This shows the wide variability in sodium content across brands and manufacturers. One highlights a similar state of affairs that indicates that the South African consumer market is awash with both compliant and non-compliant products whereby the sodium content differences across products under the same categories were wide [16]. Thus, while some products were highly compliant, for example containing as little as 5 mg Na/100 g, some were severely overstocked with sodium contents of 113 mg Na/100 g.

Looking at the different categories, the following findings were made:

- In the C1 category, 7.5% of the 40 varieties sampled did not meet their 2019 target, while 27.5% did not meet the target for 2016.
- In the C2 category, all 40 varieties met their 2019 target, while 20% did not meet their 2016 target.
- In the C3 category, 90% met their 2019 target, while 40% did not meet their 2016 target.

In the earlier cited study [16], a slightly different picture regarding meeting 2016 targets is highlighted. In that study, as of 2017, 41% of potato crisps (C2) had not met their 2016 target, while 42% of salt and vinegar snacks and crisps had also failed to meet their 2016 Na mg/100 g compliance levels. This confirms that the meeting of stipulated targets has generally been lagging. Nonetheless, when comparing these 2016 targets to this study’s findings, it is worth noting that in this study, products had at least five years to comply with the 2016 targets, giving them more adjustment time. This view can conclude that perhaps more readjustment time could lead to more compliance. All the same, it is worrisome that even after such a long readjustment time, some of the salt and vinegar-flavoured crisps and snacks still failed to comply with the 2016 sodium content regulations. Some suggest that more studies should be conducted to verify the effectiveness of sodium reduction strategies, and they should include data on sodium diet intake and changes over time in sodium levels available at retail shops [39].

C1 was the only category with five sodium-related nutrient content claims. C2 and C3 products did not make any nutrient content claim. One product in the C1 category had a sodium nutrient content claim which was not non-compliant. The labelling legislation R.146 clearly states that for a food product to be eligible to make a “low in sodium” claim, the sodium level per 100 g needs to be 120 mg or below. The product had 270 mg Na/100 g on the nutritional panel, and the lab analysed value was 380 mg Na/100 g. A designed labelling system should be designed in a way that makes it clear for consumers to understand [35]. The FOP labelling system should present key nutrients such as fat, sugar and sodium with easy-to-understand words reflecting whether the nutrient is low, medium or high.

### 4.5. Overall Compliance with 2016 and 2019 Targets

In the total sample, 96% of the varieties met their 2016 targets, while only 4% did not. In addition, 74% met their 2019 target, while 26% did not. One of the major debates surrounding mandatory sodium reduction in foodstuffs has been its effectiveness, especially whether producers and distributors of listed foodstuffs would duly comply [40]. The above results highlight that even though some of the studied products did not comply with the 2016 and 2019 regulations, government involvement in the process positively affects the extent to which producers and distributors attempt to reduce sodium content in products.

Similarly, a study which investigated the compliance of seven foodstuffs with R214′s 2016 and 2019 sodium content targets comments that there is evidence that regulation can result in reduced-sodium composition in foods [33]. The study further comments that small cases of non-compliance with both 2016 and 2019 targets among some products highlight the need for continuous monitoring and evaluation and follow-ups on manufacturers to ensure compliance.

### 4.6. Average Category Performance above 2016 and 2019 Mg Na/100 g Benchmarks

The average for the C1 variety’s Mg Na/100 g was 38% below the 2019 limit, which was followed by C3 (23%) and C2 (22%). A modelling study to evaluate replacing sodium chloride with potassium chloride using three different formulations recommended using potassium chloride in food products as a replacement for sodium chloride [41].

C1 varieties were on average 26% above the set sodium content limit, which was followed by C3 varieties that were on average 4% above the 2016 targeted limit. In Brazil, it was pointed out in a study to assess sodium levels of snacks consumed by children and adolescents out of the 2945 foods analysed. It was reported that 21% of the foods had high levels of sodium (>600 mg Na/100 g) [42]. In SA, an assessment was conducted to measure the sodium of content foods, and snack foods had the highest median level of sodium reported at (746/mg/100 g) [16].

In Slovenia, the sodium content of foodstuffs was monitored in 2015 and compared to data previously collected in 2019. The authors advised that the National Action Plan to reduce sodium intake had an inadequate impact on the pre-packaged food products, which contribute to a high sodium intake in the diets of its population. They also highlighted that effective industry collaboration and consumer education should be strengthened [43].

### 4.7. Low-Sodium Claims by Evaluated Products

C1 was the only category with products with a “low in sodium” claim. Only one of these had a lower than labelled sodium content. Others had lower sodium content on their labels than those detected in the laboratory. Under South African law, manufacturers can make three types of sodium content claims. These are “low” for products containing 120 mg Na per 100 g, “very low” for products with 40 mg Na/100 g and “Virtually free or free” for products containing 5 mg Na/100 g or less [36]. Three of the snacks (Savoury Snack 2, 3 and 4) made a “Low in sodium”, albeit, in reality, they each had 8.81 mg Na/100 g, 8.92 mg Na/100 mg and 9.47 Na/100 mg falling in the very low sodium content range. The other two snacks (Savoury Snack 1 and Savoury Snack 14) remained in the same content claim category even after factoring in the differences between recorded and observed mg Na/100 g. The researcher opines that this could result from measuring method differences rather than misleading labelling considering the small differences. The case of Savoury Snack 14 is worrisome, as the product made a low sodium content claim even after reporting a 270 mg Na/100 g, which proved to be 380 mg Na/100 mg after the ICP-AES test.

Bursey, Wiles and Biggs found a relatable case in a South African study where a product with a high sodium content above 40 mg Na/100 g came with a low sodium content claim [44]. The researchers comment that reporting inaccuracies were common and manufacturers needed to fine-tune their testing and reporting to comply with labelling regulations on sodium content and other elements [44]. 

Thus, there were considerable differences between what was reported on the product label and what was detected in laboratory tests. It was outside the scope of this study to determine the reasons or explanations behind these variations, but several studies have attempted to explain them. Product labelling is the information link between the ordinary consumer and the producer communicating a product’s constituents and their quantifications [45]. Any variances between the labelled information and the actual constituents could expose consumers to the risks and dangers they would otherwise have escaped. Some scholars attribute such differences to intentional acts of public misinformation, which might be classified as food fraud [46]. Others, however, see the use of different measuring methods as the cause of such discrepancies.

In a Spanish study, it was articulated that manufacturers can abuse nutrition claims to increase sales volumes, and authorities need to implement sufficient plans to encourage healthy eating and better food labelling strategies [47].

Nutrition claims can influence the type of products consumers buy and the consumption of certain foods [48]. Incorrect labelling can be deceitful to consumers and hinder the work of public health, and for these reasons, this is a matter that should be urgently dealt with [49].

## 5. Limitations

The study reports a few limitations. Only products which were available during data collection were bought from Durban. These products were bought from the five major food retailers: Spar, Shoprite, Checkers, Woolworths, and Pick n Pay. Products from other outlets, including those made in the informal sector, were excluded, and the study’s results do not apply to these categories. In addition, only three snack categories from the sodium regulation list were analysed as part of this study. It, therefore, excludes several categories, and its results may not apply to these excluded groups. Finally, only the products with a sodium nutrient content claim were verified through lab analysis.

## 6. Conclusions

Data from this study show that products from retail shops still do not comply with the sodium reduction legislation. There are impressive attempts by manufacturers to comply with R.214, although the risk of non-compliance remains. This risk exposes the consumer to high-sodium content snacks that have been extensively discussed as a major risk factor among South African consumers. The Africa-Predict study conducted in South Africa using 24 h urine samples showed that sodium intake by individuals aged 20–30 years decreased by 1.2 g/day after the implementation of the mandatory sodium regulations [50]. This is great progress, as previously reported research using regress equations further highlighted that by decreasing the sodium content of certain foodstuffs in South Africa, the initiative could lead to averting 7400 cardiovascular-related deaths [51].

There are various cardiovascular-related benefits of reduced sodium intake in humans. A diet low in sodium can positively affect the cardiovascular system, assisting in stable blood pressure values in hypertensive patients and can assist in vascular functioning [52]. The dietary strategy which includes low-sodium foods and Dietary Approaches to Stop Hypertension (DASH) showed significant reductions in systolic and diastolic blood pressure, respectively [53].

Another risk comes from poorly labelled products that claim to contain low-sodium levels which are not compliant with the labelling legislation. This exposes consumers to misinformed decision making that also adds to the risk that low-sodium-seeking consumers could face when buying savoury snacks. While there is general compliance with R.214, there is also evidence that this regulation’s set targets fall far above WHO (2021) sodium levels for a variety of food categories [54].The study, therefore, concludes that despite a generally higher compliance rate, more action needs to be taken to ensure that the population is consuming acceptable levels of sodium. 

## 7. Recommendations

Various recommendations were made for food manufacturers, the government and consumers, and these stem from the empirical research and the literature consulted. These are outlined in this section.

- Manufacturers needed to look at possible replacements for salt in savoury snacks. In the literature, it was suggested that green salt, lemon and vinegar were among the effective flavour enhancers that could be used in manufacturing.
- Food manufacturers should ensure a sodium testing programme to identify discrepancies in sodium content reporting on food labels.
- Manufacturers are encouraged to enhance their sodium content reporting and labelling systems to comply with government needs. This is critical, as some products in the study had incorrect sodium content information.

The following recommendations apply to the government:

- The government should develop a sodium reduction monitoring plan that the public can access to enforce regulation and ensure industry compliance.
- There is a gap in what the DoH is doing regarding food manufacturers not complying with the regulation. More visibility is needed regarding what the department does to ensure manufacturers comply with the regulation.
- The DoH should consider implementing mandatory FOP labelling systems, which will be easy for consumers to understand, as South Africa is diverse. This could include the easy-to-read traffic light system used by the WHO.
- The DoH should have consumer awareness campaigns on radio, TV, and social media apps to raise awareness of the effects of a diet high in salt intake and highlight the benefits of using alternative ingredients when consumers are preparing meals at home.
- The government needs to consider further cutting the sodium targets in salty savoury snacks to below WHO recommended standards that put any Na content above 600 mg Na/100 g.

Consumers and consumer groups need to keep abreast with salt and sodium content developments to stay informed on the risks and dangers associated with sodium as a food substance. They also need to work toward protecting the child consumer reported in the literature as a major at-risk group due to their high consumption of salty, savoury snacks. Consumer groups need to lobby the government and manufacturers to reveal non-compliance among products and publicly report the risks associated with non-compliant snack products.

## Figures and Tables

**Table 1:** Reduction in total sodium content (Na) of certain foodstuffs (Source: Department of Health, 2013).

Product	2016 Target (Na/100 mg)	2019 Target (Na/100 mg)
C1: Ready-to-Eat Savoury Snacks	800	700
C2: Flavoured Potato Crisps	650	550
C3: Flavoured Ready-to-Eat, Savoury Snacks and Potato Crisps—Salt and Vinegar Only	1000	850

**Table 2:** Product performance against Na/100 mg benchmarks or targets for 2016 and 2019.

	C1: Ready-to-Eat Savoury Snacks (*n* = 40)	C2: Flavoured Potato Crisps (*n* = 40)	C3: Flavoured Ready-to-Eat, Savoury Snacks and Potato Crisps—Salt and Vinegar Only (*n* = 10)
*n*	40	40	10
2016 Target (Na/100 g)	800	650	1000
2019 Target (Na/100 g)	700	550	850
Mean	532.33	506.68	798.6
Median	595	519	848
Std. Deviation	278.79	68.69	188.54
Skewness	−0.40	−0.53	−0.39
Kurtosis	−0.03	0.25	−1.30
Range	1108	305	524
Minimum	5	325	512
Maximum	1113	630	1036
Missed 2016 targets	7%	0%	10%
Missed 2019 targets	27%	20%	40%

**Table 3:** Descriptive summary—all snacks.

		Valid	Target (Na/100 g)	Mean	Median	SD	Skew.	Kurt.	Range	Min.	Max.
RTE Snacks (C1)	Met 2016 target	37	800	493.62	585	248.8	−0.9	−0.49	788	5	793
	Did not meet 2016 target	3	800	1009.7	1113	179	−1.7	0	310	803	1113
	Met 2019 target	29	700	424.38	547	237.4	−0.8	−0.99	690	5	695
	Did not meet 2019 target	11	700	816.91	758	149.7	1.7	1.6	406	707	1113
Flavoured Potato Crisps (C2)	Met 2016 target	40	650	506.68	519	68.7	−0.5	0.2	305	325	630
	Did not meet 2016 target	0	650	0	0	0	0	0	0	0	0
	Met 2019 target	32	550	485.22	500.5	58.2	−1	0.6	225	325	550
	Did not meet 2019 target	8	550	592.5	597.5	28	−0.072	−1.8	73	557	630
Salt and Vinegar Snacks (C3)	Met 2016 target	9	1000	772.22	846	179.34	−0.33	−1.44	475	512	987
	Did not meet 2016 target	1	1000	1036	1036	0	0	0	0	1036	1036
	Met 2019 target	6	850	685.83	674.5	149.92	0.07	−2.47	338	512	850
	Did not meet 2019 target	4	850	967.75	987	74.82	−1.41	2.65	175	861	1036

**Table 4:** Varieties failing to meet both 2016 and 2019 targets.

	Sodium Level per 100 g on Pack	2016-Sodium Limit per 100 g (mg Na)	2019-Sodium Limit per 100 g (mg Na)	2016 Target vs. Current	2019 Target vs. Current
Savoury Snack 24	1113	800	700	−313	−413
Savoury Snack 25	1113	800	700	−313	−413
Savoury Snack 29	803	800	700	−3	−103
Salt and Vinegar Snack 1	1036	1000	850	−36	−186

## Data Availability

Data sets are available upon request.
